# Determinants of Entero-Invasive and Non-Entero-Invasive Diarrheagenic Bacteria Among HIV-Positive and HIV-Negative Adults in Ghana

**DOI:** 10.3390/medsci13040316

**Published:** 2025-12-12

**Authors:** Hagen Frickmann, Fred Stephen Sarfo, Betty Roberta Norman, Albert Dompreh, Shadrack Osei Asibey, Richard Boateng, Veronica Di Cristanziano, Tafese Beyene Tufa, Ulrike Loderstädt, Ramona Binder, Andreas Erich Zautner, Tom Luedde, Torsten Feldt, Kirsten Alexandra Eberhardt

**Affiliations:** 1 Department of Microbiology and Hospital Hygiene, Bundeswehr Hospital Hamburg, 22049 Hamburg, Germany; frickmann@bnitm.de; 2 Institute for Medical Microbiology, Virology and Hygiene, University Medicine Rostock, 18057 Rostock, Germany; 3 Department of Medicine, Komfo Anokye Teaching Hospital, Kumasi 00233, Ghana; 4 Department of Internal Medicine, School of Medical Sciences, Kwame Nkrumah University of Science and Technology, Kumasi 00233, Ghana; 5 Department of Clinical Microbiology, Komfo Anokye Teaching Hospital, Kumasi 00233, Ghanarichardboateng166@gmail.com (R.B.); 6 Institute of Virology, Faculty of Medicine and University Hospital Cologne, University of Cologne, 50937 Cologne, Germany; 7 Asella Referral and Teaching Hospital, College of Health Sciences, Arsi University, Asella P.O. Box 04, Ethiopia; 8 Hirsch Institute of Tropical Medicine (HITM), Heinrich Heine University, Asella P.O. Box 04, Ethiopia; torsten.feldt@med.uni-duesseldorf.de; 9 Department of Gastroenterology, Hepatology and Infectious Diseases, University Hospital and Medical Faculty of the Heinrich Heine University, 40225 Düsseldorf, Germany; 10 Institute of Infection Control and Infectious Diseases, University Medical Center Göttingen, 37075 Göttingen, Germany; 11 Laboratory Medicine and Transfusion Medicine Department, IMD Laboratory Greifswald, 17493 Greifswald, Germany; 12 Institute of Laboratory Medicine and Microbiology & Vaccination Center, Klinikum Würzburg Mitte, Campus Juliusspital, 97070 Würzburg, Germany; azautne@gwdg.de; 13 Institute of Medical Microbiology and Hospital Hygiene, Medical Faculty, Otto-von-Guericke University Magdeburg, 39120 Magdeburg, Germany; 14 Department of Tropical Medicine, Bernhard Nocht Institute for Tropical Medicine, 20359 Hamburg, Germany; 15 I. Department of Medicine, University Medical Center Hamburg-Eppendorf, 20246 Hamburg, Germany

**Keywords:** *Salmonella*, *Shigella*, *Campylobacter*, enteropathogenic, enterotoxigenic, enteroaggregative, *Escherichia coli*, *Arcobacter butzleri*, immunosuppression, HIV

## Abstract

Objectives: This observational and cross-sectional study investigated differential associations between entero-invasive and non-entero-invasive enteric pathogens and HIV infection, considering socioeconomic, clinical and immunological aspects. In a Ghanaian population with a high prevalence of enteric pathogens, stool samples from people living with HIV (PLWH) were screened for *Salmonella* spp., *Shigella* spp./EIEC (enteroinvasive *Escherichia coli*), and *Campylobacter jejuni* as entero-invasive bacteria, for enteropathogenic *E. coli* (EPEC), enterotoxigenic *E. coli* (ETEC), and enteroaggregative *E. coli* (EAEC) as non-entero-invasive bacteria. *Arcobacter butzleri*, with uncertain enteropathogenicity, was also included. Methods: Stool samples from PLWH (with and without antiretroviral therapy) and HIV-negative controls were analyzed by real-time PCR for the presence and quantity of the selected enteropathogens. Results were correlated with socioeconomic, clinical, and immunological parameters. Results: The presence of *Shigella* spp. /EIEC in stool was both qualitatively and quantitatively associated with reduced CD4+ T lymphocyte counts and was qualitatively associated with clinically apparent diarrhea. EAEC showed a weak positive association with HIV infection, supported by a negative correlation between EAEC DNA quantity and CD4+ T lymphocyte counts. EPEC colonization was associated with HIV negativity, higher CD4+ T lymphocyte counts, and lower socioeconomic status. Abundance of *Salmonella enterica* was associated with clinically apparent diarrhea. Conclusions: This explorative, hypothesis-forming study suggests species- or pathovar-specific associations between enteric bacterial pathogens and HIV-related immunosuppression. Observed relationships with clinically apparent diarrhea largely align with findings from sub-Saharan African children, except for a more pronounced association between diarrhea and *Salmonella* in this cohort.

## 1. Introduction

Infections with several enteric pathogens have been reported as more frequent in patients with acquired immunodeficiency syndrome (AIDS), which results from infection with the human immunodeficiency virus (HIV). Among bacterial microorganisms causing entero-invasive disease in susceptible individuals, the strongest associations have been documented for *Salmonella* spp. (including non-typhoidal serovars), *Shigella* spp., and *Campylobacter jejuni* [1,2,3,4,5]. Increasing antimicrobial resistance rates in these pathogens, particularly in tropical regions with high endemicity, complicate therapeutic management [6]. Even independent of acquired resistance, salmonellosis, shigellosis, and campylobacteriosis in patients with HIV often show therapeutic failure or recurrence [7].

Besides common fecal–oral transmission, sexual transmission via the anal–oral route has been well documented for *Shigella* spp. [8] and thermophilic *Campylobacter* spp. [9]. Concomitant HIV infection has been identified as a risk factor for shigellosis [8,10]. While dysentery is the typical manifestation of shigellosis, *Shigella* spp. bacteremia is strongly associated with immunosuppression due to HIV infection [11], and persistent or chronic *Shigella*-associated enteritis has been observed in immunocompromised patients [12].

AIDS increases *Salmonella* spp. infection risk approximately 20-fold. Bacteremia occurs in about 40% of co-infected patients [7]. The link between non-typhoidal *Salmonella* (NTS) bacteremia and HIV infection is particularly strong [4,13,14]. In addition to malnutrition, malaria, anemia, sickle cell disease, and young age, HIV infection is an established risk factor for systemic NTS infection [15,16,17,18]. Reported focal manifestations of disseminated NTS in patients with HIV include difficult-to-treat infections such as endocarditis [19], liver abscess [20], and meningitis [21]. Impaired macrophage function drives NTS invasiveness in HIV patients [22]. Strain-specific traits, such as enhanced intracellular survival and modulation of immune cell migration, likely contribute to long-term intestinal persistence and prolonged bacterial shedding [23,24].

The association between invasive campylobacteriosis and HIV infection is also well established [25,26,27]. In individuals with HIV, *Campylobacter jejuni* bacteremia can cause severe febrile illness with poor response to antibiotic therapy, recurrent or prolonged courses, extra-intestinal involvement (e.g., pulmonary or dermatological), and elevated mortality [26]. In contrast, infections in immunocompetent individuals are typically self-limiting and respond well to antibiotics [26]. Similarly to shigellosis, sexual transmission of *Campylobacter* spp. via the anal–oral route has also been confirmed [7,28,29].

Likely sexual transmissibility has been proposed for diarrheagenic *Escherichia coli* [30] and other enteric bacteria [31] as well. However, consistent associations between HIV infection and non-invasive *E. coli* pathovars such as enteropathogenic (EPEC), enterotoxigenic (ETEC), or enteroaggregative (EAEC) *E. coli* have not been demonstrated convincingly. These pathovars commonly occur in both HIV-positive and HIV-negative individuals, with or without diarrheal disease, especially in high-endemicity regions [32,33,34,35,36]. For example, a recent Iranian study found thalassemia more strongly associated with diarrheagenic *E. coli* than HIV positivity [32]. Nevertheless, HIV infection may facilitate colonization or infection with diarrheagenic *E. coli* in sub-Saharan Africa [34], whereas such associations have not been confirmed in industrialized settings [36].

*Arcobacter butzleri* (homotypic synonym *Aliarcobacter butzleri*) is a bacterial species frequently detected in human stool samples and associated with gastroenteritis, abdominal pain, and acute or persistent watery diarrhea [37]. Adhesive, cytotoxic, and invasive properties of *A. butzleri* contribute to its pathogenic potential [37,38,39,40,41,42], with epithelial barrier dysfunction likely leading to leak-flux-type diarrhea [43]. In immunocompromised individuals, including those with HIV infection, *A. butzleri* and other *Arcobacter* spp. have also been reported to cause invasive disease and bacteremia [44,45]. Similarly, patients with other severe conditions predisposing them to bacterial translocation, such as liver cirrhosis, may develop *A. butzleri* bacteremia [46]. Despite these reports [44,45], a recent epidemiological study in sub-Saharan Africa did not confirm a significant association between HIV infection and *A. butzleri* prevalence in stool samples [47]. An Indian study also found detection rates too low for meaningful conclusions [48].

In this study, *Salmonella* spp., *Shigella* spp./EIEC, and *C. jejuni* were selected as representative entero-invasive bacterial pathogens with known associations to HIV infection. EPEC, ETEC and EAEC were included as non-invasive enteropathogens with unclear HIV correlation, and *A. butzleri* was investigated as a potentially invasive bacterium under conditions of immunosuppression. Ghana was chosen as a study site owing to its considerable HIV prevalence of approximately 1.6% [49]. As a resource-limited tropical country, where maintaining high standards of food hygiene is challenging, it is also significantly burdened by gastroenteric infections. At the time of sampling for our study, the prevalence of gastroenteric pathogens in Ghana was primarily derived from cross-sectional epidemiological studies conducted in children. One study reported prevalence values exceeding 50% for ETEC, over 30% for *Shigella* spp. and *Campylobacter* spp., and greater than 10% for *Salmonella* spp. in Ghanaian children, regardless of symptomatic presentation [50].

The study aimed to assess the differential associations of these invasive and non-invasive enteropathogens with socio-economic, clinical, and immunological characteristics of Ghanaian HIV patients, thereby contributing to understanding their etiological relevance in immunocompromised individuals.

## 2. Materials and Methods

### 2.1. Study Design and Setting

People living with HIV (PLWH) attending the outpatient HIV clinic at Komfo Anokye Teaching Hospital in Kumasi, Ghana, were invited to participate in this cross-sectional, observational, hypothesis-generating study. Even beyond the present study, the research focused on exploring associations between gastrointestinal and other pathogens and various socio-demographic, clinical, and immunological factors [51,52]. As a control group, HIV-negative adults from the same region were also enrolled. Equal numbers of PLWH receiving and not receiving antiretroviral therapy ensured the assessment of possible therapy effects. Notably, the samples were collected between November 2011 and November 2012, thus before the publication of the START study group findings, which ultimately led to the recommendation to initiate antiretroviral combination therapy as soon as possible [53]. The entire cohort, comprising both HIV-positive and HIV-negative participants, was recruited over a 12-month period. Demographic, socio-economic, immunological, and clinical data were collected via standardized questionnaires administered by trained study personnel. Given that the study included only native Ghanaians living under regional conditions throughout their lives, and since the assessment was conducted outside of an outbreak situation, more specific questions regarding consumption of food from restaurants or vendors were deemed unlikely to yield conclusive results.

### 2.2. Laboratory Diagnostics

Venous blood samples were collected to determine CD4+ T lymphocyte counts using a FACSCalibur flow cytometer (Becton Dickinson, Mountain View, CA, USA) in Ghana. HIV-1 viral loads were quantified with a Real-Time HIV-1 PCR system (Abbott Diagnostics, Wiesbaden, Germany). Peripheral blood mononuclear cells (PBMCs) were isolated from heparinized venous blood by Ficoll/Hypaque (Biocoll Separating Solution, Biochrom AG, Berlin, Germany) density gradient centrifugation, washed with phosphate-buffered saline, and resuspended in RPMI 1640 medium (Gibco Invitrogen, Carlsbad, CA, USA) with heat-inactivated fetal calf serum (Biochrom AG, Berlin, Germany). Cells were cryopreserved in liquid nitrogen, shipped to Germany, stained for immune activation markers, and analyzed by LSRII flow cytometry (BD Biosciences, Heidelberg, Germany) using FlowJo (v9.6.2, Tree Star, San Carlos, CA, USA).

Native stool aliquots were stored at −80 °C until DNA extraction, which was performed according to the manufacturer’s instructions using the QIAamp stool DNA mini Kit (Qiagen, Hilden, Germany). In-house established laboratory-developed real-time PCR assays as reported in the literature [54,55,56,57] were used to assess the samples for *Salmonella* spp., *Shigella* spp./enteroinvasive *E. coli* (EIEC) (not further discriminable due to the use of a shared target sequence), *C. jejuni*, enteropathogenic *E. coli* (EPEC), enterotoxigenic *E. coli* (ETEC), enteroaggregative *E. coli* (EAEC) and *A. butzleri*. In more detail, the assays targeted a 180-base pair sequence of the *ttr*C gene of *Salmonella* spp., a 170-base pair sequence of the *ipa*H gene of *Shigella* spp./EIEC, a 249-base pair sequence of the *gyr*A gene of *C. jejuni*, a 102-base pair sequence of the *eae* gene of EPEC, a 76-base pair sequence of the EAF plasmid of EPEC, a 68-base pair sequence of the *elt*A gene of ETEC, a 251-base pair sequence of the *est*B gene of ETEC, a 105-base pair sequence of the *aat*A gene of EAEC, and a 170-base pair sequence of the *rpo*B/C gene of *A. butzleri*, respectively. Of note and as shown in Appendix A Table A1, the *elt*B and *est*B genes of ETEC were jointly assessed in the same fluorescence channel, resulting in a common cycle threshold (Ct) value. In contrast, the *eae* gene and the EAF plasmid of EPEC were assessed in different fluorescence channels. Positivity of a sample for EPEC was accepted if at least one out of the two parameters provided a positive real-time PCR signal. As described in detail elsewhere [54,57,58], the assays’ diagnostic accuracy was estimated with sensitivities in the 61–100% range, specificities in the 97–100% range, and limits of detection in the 1.2 × 10^1^–3.7 × 10^2^ copies per µL eluate range. Appropriate amplification conditions in each real-time PCR run were monitored with a PCR grade water-based negative control and a positive control consisting of a plasmid containing the respective target sequence inserted in a pEX-A128 vector backbone. Appendix A Table A1 provides details on both the applied oligonucleotides and the limits of detection of the used real-time PCR assays.

Corbett Q cyclers (Qiagen, Hilden, Germany) were used to run the above-described real-time PCR assays. The master mix compositions and run conditions of each real-time PCR assay are shown in detail in Appendix A Table A2. Of note, a Phocid herpesvirus (PhHV) DNA-specific real-time PCR described elsewhere [59] was used to control sample inhibition.

### 2.3. Statistics

A socioeconomic status (SES) index was derived using principal component analysis (PCA) based on three binary household indicators: access to tap water, availability of a refrigerator, and electricity. The first principal component was used as the SES index, and the median value of this index was used to classify participants as having low or high SES. Descriptive analyses used Fisher’s exact test for categorical variables. The Spearman rank correlation coefficient (ρ), chosen because it does not require normality assumptions and is appropriate for typically skewed Ct values, quantified the strength of the relationship between continuous variables. Due to the high prevalence of coinfections with multiple bacterial enteropathogens in both HIV-positive and HIV-negative individuals, traditional univariate analyses examining individual pathogens separately may overlook the combined and potentially interacting effects of coinfections on clinical, immunological, and socioeconomic outcomes. To address this complexity, we applied a machine learning approach—random forest analysis (500 trees, 2 variables tried per split)—conducted separately in HIV-positive and HIV-negative groups. This methodology accommodates multiple correlated predictors, identifies key enteropathogens most strongly associated with CD4+ T cell counts, diarrheal disease, and SES, and captures nonlinear relationships. Missing data were handled by complete-case analysis (no imputation). Variable importance was quantified by the random forest algorithm using either the Increase in Node Purity (for continuous outcomes) or the Mean Decrease in Gini (for binary outcomes), reflecting each predictor’s relative contribution to model performance. Higher values indicate stronger influence on prediction accuracy. Following random forest prioritization, multivariable regression models were fitted comprehensively on the entire cohort: linear regression for continuous outcomes and logistic regression for binary outcomes. No sensitivity analyses were performed, and due to the exploratory, hypothesis-forming nature of the present study, correction for multiple testing (e.g., Bonferroni) was not applied [60]. Statistical analyses were conducted using the software R (version 4.4.3, R Foundation for Statistical Computing, Vienna, Austria). All statistical tests were two-sided with a significance threshold of *p* < 0.05.

### 2.4. Ethics

The study was conducted in accordance with the Declaration of Helsinki and its amendments. Sample collection and analysis followed protocols approved by the Committee on Human Research at Kwame Nkrumah University of Science and Technology, Kumasi, Ghana (approval no. CHRPE/AP/82/11, dated 8 September 2011), and the Ethics Committee of the Medical Council in Hamburg, Germany (approval no. PV3771, dated 13 May 2011). Written informed consent was obtained from all participants prior to enrollment.

## 3. Results

### 3.1. Study Population

Stool samples were available from 651 HIV-positive and 84 HIV-negative individuals (Table 1). The median age was 40 years (IQR 33–47) among HIV-positive participants and 29 years (IQR 24–37) among HIV-negatives, with females comprising 73.6% and 64.6%, respectively. Over half of participants had access to tap water, and most had electricity and refrigerators in their households. The socioeconomic status index indicated that approximately three-quarters of HIV-positive participants had a high SES, comparable to the HIV-negative group. Clinically, one-third of HIV-positive participants were on cotrimoxazole prophylaxis, and 41.3% received combination antiretroviral therapy. The median body mass index was slightly lower in HIV-positive individuals (22; IQR 20–26) than in HIV-negative individuals (24; IQR 21–27). Immunologically, HIV-positive individuals showed a median viral load of 4.2 log10 copies/mL (IQR 1.6–5.4), lower median CD4+ T lymphocyte counts (347 cells/µL; IQR 145–571) versus HIV-negative individuals (949 cells/µL; IQR 767–1168), higher CD8+ T lymphocyte counts (970 cells/µL; IQR 648–1379 vs. 470 cells/µL; IQR 354–710), and reduced CD4+/CD8+ ratios (0.4; IQR 0.2–0.7 vs. 2.0; IQR 1.6–2.5).

### 3.2. Detection of Bacterial Enteropathogens in Stool Samples According to HIV Infection

**Figure 1:**
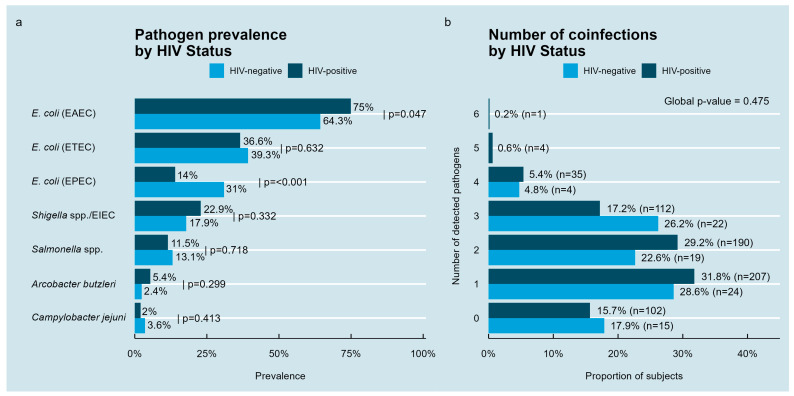
DNA-detection-based prevalence and absolute numbers of coinfections with enteropathogenic bacteria by HIV status in the study population. (**a**) Prevalence of detected enteric pathogens in stool samples, stratified by HIV-negative and HIV-positive groups. (**b**) Distribution of the number of concurrent detected enteric bacterial infections according to HIV status.

Prevalence of enteric bacterial pathogens was assessed separately in HIV-positive (n = 651) and HIV-negative (n = 84) individuals (Figure 1a). Among HIV-negative participants, enteroaggregative *E. coli* (EAEC) was most prevalent at 64.3% (n = 54), followed by enterotoxigenic *E. coli* (ETEC) at 39.3% (n = 33) and enteropathogenic *E. coli* (EPEC) at 31.0% (n = 26). *Salmonella* spp. and *Shigella* spp./enteroinvasive *E. coli* (EIEC) were detected in 13.1% (n = 11) and 17.9% (n = 15), respectively, while *A. butzleri* and *C. jejuni* were rare (2.4% [n = 2] and 3.6% [n = 3]). In HIV-positive individuals, *E. coli* (EAEC) prevalence was higher at 75.0% (n = 488), followed by *Shigella* spp. (22.9%, n = 149), which was more prevalent among those not receiving ART (*p* = 0.053), EPEC (14.0%, n = 91), *Salmonella* spp. (11.5%, n = 75), *A. butzleri* (5.4%, n = 35), and *C. jejuni* (2.0%, n = 13). EAEC was significantly more prevalent in HIV-positives (*p* = 0.047), while EPEC was more common in HIV-negatives (*p* < 0.001); within PLWH, EAEC was more frequent in those not receiving ART (*p* = 0.002). No significant differences were observed for other pathogens.

Coinfection analysis stratified by HIV status (Figure 1b) showed that 82.1% (n = 69) of HIV-negative individuals and 84.3% (n = 549) of HIV-positive individuals harbored detectable enteric pathogen DNA. Among HIV-negative individuals, most had one (28.6%, n = 24), two (22.6%, n = 19), or three (26.2%, n = 22) simultaneous infections. A smaller fraction (4.8%, n = 4) had four coinfections, with no cases exceeding that count. In contrast, among HIV-positives, 31.8% (n = 207), 29.2% (n = 190), and 17.2% (n = 112) had one, two, or three coinfections, respectively. A total of 5.4% (n = 35) had four concurrent infections, with rare cases of five (0.6%, n = 4) and six (0.2%, n = 1) infections in parallel.

### 3.3. Enteric Pathogens Associated with CD4+ T-Lymphocyte Counts

**Figure 2:**
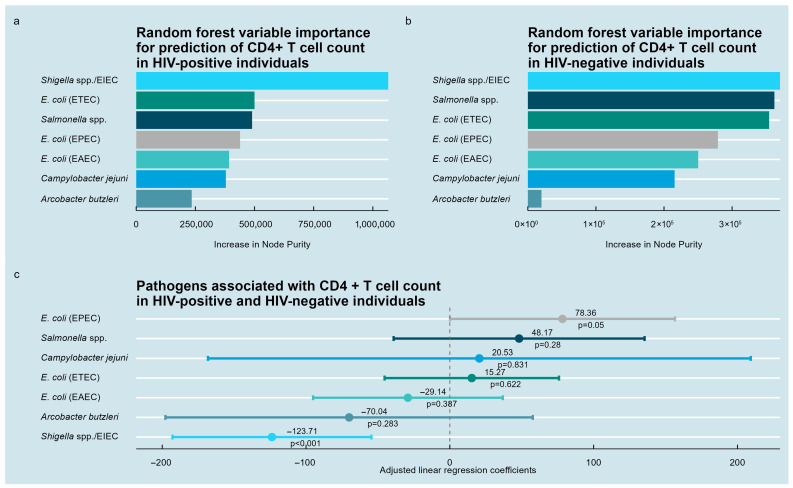
Pathogens associated with CD4+ T-lymphocyte counts in the study population. (**a**) Random forest variable importance analysis for HIV-positive individuals identifying bacterial enteropathogens ranked by their predictive contribution to CD4+ T-lymphocyte counts. (**b**) Corresponding random forest analysis for HIV-negative individuals. (**c**) Multivariable linear regression results for the combined cohort showing estimated associations between pathogens and CD4+ T-lymphocyte counts.

Random forest analysis, conducted separately for HIV-positive and HIV-negative individuals, identified the relative importance of bacterial pathogens in predicting CD4+ T-lymphocyte counts (Figure 2a,b). In HIV-positive participants, *Shigella* spp./EIEC exhibited the highest importance, followed by *Salmonella* spp., ETEC, EPEC, EAEC, *C. jejuni*, and *A. butzleri*. Among HIV-negative participants, *Shigella* spp., *Salmonella* spp., and ETEC ranked highest.

Multivariable linear regression on the entire cohort revealed few statistically significant associations with CD4+ T-lymphocyte counts. *Shigella* spp. was independently associated with a lower CD4+ T-lymphocyte count (β = −124, 95% CI: −193 to −54.5, *p* < 0.001). EPEC showed a robust trend toward positive association (β = 78.4, 95% CI: 0.1 to 157, *p* = 0.05). Associations with *Salmonella* spp., *C. jejuni*, *A. butzleri*, ETEC, and EAEC were not significant (Figure 2c).

Additional analyses of other immune parameters in the HIV positive subgroup revealed patterns broadly consistent with these findings: *Shigella* spp. presence was linked to lower CD4 counts (β = −96.3, *p* = 0.002) and lower CD4/CD8 ratio (β = −0.116, *p* = 0.032), consistent with immunosuppression. *Shigella* spp. were also associated with higher HLA-DR+CD38+CD8+ cells (β = 6.17, *p* = 0.042), indicating CD8 activation.

### 3.4. Pathogens Associated with Diarrheal Disease

**Figure 3:**
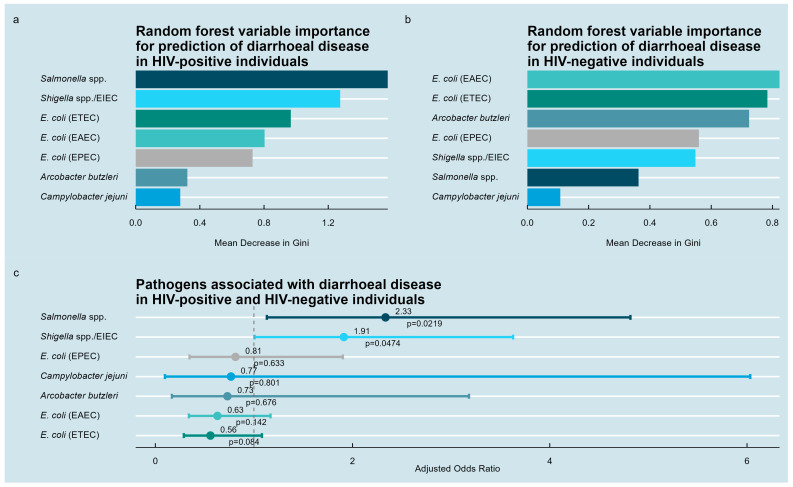
Pathogens associated with diarrheal disease in the study population. (**a**) Random forest variable importance analysis for HIV-positive individuals identifying enteropathogenic bacteria ranked by their predictive contribution to the clinical symptom of diarrhea. (**b**) Corresponding random forest analysis for HIV-negative individuals. (**c**) Multivariable logistic regression results for the entire study population showing estimated associations between pathogens and diarrheal disease.

To identify bacterial pathogens associated with diarrheal disease, random forest analyses were performed separately for the HIV-positive and HIV-negative subgroups (Figure 3a,b). Among HIV-positive participants, *Salmonella* spp. exhibited the highest importance, followed by *Shigella* spp., ETEC, EAEC, EPEC, *A. butzleri*, and *C. jejuni*. In the HIV-negative group, EAEC, ETEC, *A. butzleri*, EPEC, *Shigella* spp./EIEC, *Salmonella* spp., and *C. jejuni* ranked in decreasing order.

Multivariable logistic regression combining all participants revealed statistically significant positive associations between diarrheal disease and *Salmonella* spp. (OR = 2.33, 95% CI: 1.13–4.82, *p* = 0.022) and *Shigella* spp. (OR = 1.91, 95% CI: 1.01–3.63, *p* = 0.047). No significant associations were observed for *A. butzleri*, *C. jejuni*, ETEC, EAEC, or EPEC (Figure 3c).

Notably, cotrimoxazole prophylaxis showed no relevant associations with enteric pathogen detection.

### 3.5. Pathogens Associated with the Socioeconomic Status Index

As described in the Methods section, a binary SES index was derived using principal component analysis (PCA) based on household access to tap water, availability of a refrigerator, and electricity.

**Figure 4:**
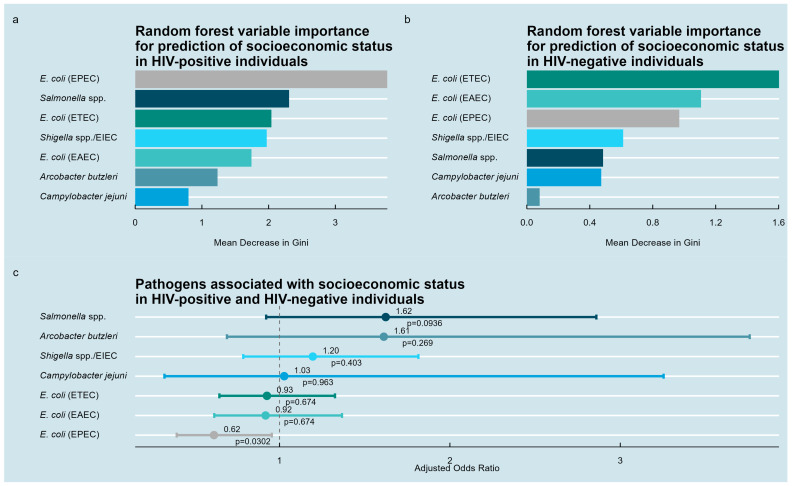
Pathogens associated with socioeconomic status index in the study population. (**a**) Random forest variable importance analysis for HIV-positive individuals identifying bacterial enteropathogens ranked by their predictive contribution to the binary socioeconomic status. (**b**) Corresponding random forest analysis for HIV-negative individuals. (**c**) Multivariable logistic regression results for the entire study population showing estimated associations between detected enteropathogens and socioeconomic status index.

Random forest analyses conducted separately in HIV-positive and HIV-negative individuals identified enteropathogenic bacteria predictive of the SES (Figure 4a,b). Among HIV-positive participants, EPEC demonstrated the highest importance, followed by *Salmonella* spp., ETEC, *Shigella* spp., EAEC, *A. butzleri*, and *C. jejuni*. In HIV-negative participants, ETEC, EAEC, EPEC, *Shigella* spp., *Salmonella* spp., *C. jejuni*, and *A. butzleri* ranked in decreasing order of importance.

Multivariable logistic regression on the entire cohort showed that only EPEC was significantly inversely associated with a high SES index (odds ratio = 0.62; 95% CI: 0.40–0.96, *p* = 0.03). No other pathogens showed statistically significant associations (Figure 4c).

### 3.6. Correlations of Cycle Threshold (Ct) Values with CD4+ T-Lymphocyte Count

Spearman rank correlations were analyzed between Ct values from real-time PCR assays targeting bacterial pathogens and CD4+ T lymphocyte counts (Table 2). Ct values for *Shigella* spp. (rho = 0.18, *p* = 0.025) and EAEC (rho = 0.10, *p* = 0.018) showed weak but statistically significant positive correlations with CD4+ T-lymphocyte counts, indicating that higher Ct values (i.e., lower pathogen loads) tend to be associated with higher CD4+ T-lymphocyte counts. Other assessed pathogens did not exhibit statistically significant correlations with CD4+ T lymphocyte counts.

## 4. Discussion

The study aimed to assess the associations between selected invasive and non-invasive enteropathogenic bacteria, as well as *A. butzleri*, and key socioeconomic, clinical, and immunological characteristics of Ghanaian individuals with and without HIV infection. The HIV-negative control group was moderately younger and had a slightly higher body mass index than HIV-positive participants, who showed the expected signs of immunosuppression. Otherwise, the two subpopulations were broadly comparable.

Concerning the overall association between enteric bacterial pathogens and HIV status, no significant links were observed for the classical enteroinvasive pathogens despite well-established evidence from earlier studies [1,2,3,4,5]. One plausible explanation is antiretroviral therapy use by approximately 41% of the HIV-positive patients, which improves immune competence by increasing CD4+ T lymphocyte counts. This likely attenuated the aforementioned associations [1,2,3,4,5], which were mostly described for HIV patients without effective antiretroviral therapy. When reduced immunocompetence, as indicated by low CD4+ T-lymphocyte counts, was considered, impaired immunity showed the expected correlation with *Shigella* spp./EIEC [1,2,3,4,5]. Multivariable analyses confirmed *Shigella* spp./EIEC presence was associated with immunosuppression markers, including lower CD4/CD8 ratios and elevated CD8 T cell activation (HLA-DR+CD38+), consistent with pathogen-driven immune responses during impaired immunity. However, no comparable pattern was observed for *Salmonella* spp. or *C. jejuni*. Importantly, the association for *Shigella* spp./EIEC was reflected not only in qualitative presence but also quantitatively, as higher enteric bacterial loads corresponded to lower CD4+ T-lymphocyte counts.

In contrast, EAEC was more frequently detected among PLWH, whereas EPEC was more common in stool samples from individuals without HIV infection. This finding differs from earlier studies reporting no such associations [32,33,34,35,36]. Prior research has linked EAEC infections to growth impairment in children and has suggested that EAEC may act as a copathogen with pathogenic importance in enteric illnesses [61,62,63,64]. Although statistical significance for EAEC approached the conventional 5% threshold, an additional correlation between higher EAEC load and lower CD4+ T-lymphocyte counts suggests the pattern may not be incidental.

The predominance of EPEC among HIV-negative individuals remains unexplained. This inverse association—EPEC presence alongside higher CD4+ T-lymphocyte counts—supports the observed trend but should be interpreted cautiously, as HIV status and CD4+ T-lymphocyte levels are not mutually independent. In line with expectations for fecal–oral transmitted organisms, EPEC detection was also associated with lower socioeconomic status across the study cohort, while no comparable associations were observed for other pathogens. These findings point to preferential EPEC colonization in socioeconomically disadvantaged individuals despite lacking evidence for additional pathology. Stable immunological control might facilitate such colonization, whereas immunosuppression may diminish it, particularly under high exposure conditions. However, the present study was not specifically designed or powered to confirm this hypothesis.

Multiple pathogen detections within individual stool samples were common in both groups, consistent with reports from other resource-limited, high-endemicity settings [50,65]. No apparent predominance of coinfections was observed in either HIV-positive or HIV-negative participants.

Regarding clinical symptoms, diarrheal disease was associated with *Salmonella* spp. and *Shigella* spp./EIEC in this high HIV-prevalence cohort, whereas no such links emerged for the remaining pathogens. As reported previously and illustrated in Table 3, the weak association between detected gastrointestinal pathogens and overt diarrheal symptoms has frequently been observed in sub-Saharan African populations, where repeated exposures are thought to induce immunological tolerance to otherwise pathogenic microorganisms [66,67]. This phenomenon is particularly well described for *Campylobacter* spp. infections [68,69], although detailed mechanisms, including potential immunological and microbiome-related contributions, remain to be elucidated [70]. Notably, the compared studies [66,67] on Ghanaian and Tanzanian children comprised higher numbers of cases and controls (approximately 2000 children each) and more than 30,000 assessed stool samples in the Tanzanian assessment. Although these historic studies focused on young children rather than adults with HIV, the similarity of the results is nevertheless striking.

In detail, comparing associations between diarrheal disease and detected microorganisms in this Ghanaian population with high HIV prevalence to previous studies among Ghanaian and Tanzanian children reveals marked similarity in the observed association patterns, with the exception of *Salmonella* spp. [66,67]. The increased virulence of *Salmonella* spp. in PLWH is consistent with earlier findings [4,7,13,14]. In contrast, the association of *Shigella* spp./EIEC with diarrhea is well documented among Ghanaian children [66], and at least bacterial load–dependent associations have been reported in Tanzanian children [67]. Similarly to *C. jejuni*, *A. butzleri* was not associated with clinically apparent diarrhea in the present sub-Saharan African study population. This finding aligns with previous observations indicating no significant association between HIV infection and *A. butzleri* occurrence in the same setting [47]. Collectively, the results suggest that recorded associations between HIV infection and bacterial causes of diarrheal disease are likely species specific, although confirmatory assessments are required to confirm or exclude causal effects.

This study has several limitations. First, its retrospective design prevents causal inference. Second, the sample size was determined by the availability of stored specimens rather than formal power calculations based on expected effect sizes. Accordingly, the analysis should be regarded as exploratory and hypothesis-generating. Third, reliance on molecular diagnostic methods precludes conclusions about viability of detected bacterial organisms. Fourth, low prevalence of some pathogens limits interpretability of non-significant associations. Fifth, suboptimal sample storage and transport conditions precluded culture-based diagnostic approaches in addition to molecular diagnosis. Notably, DNA might have been partly degraded despite −80 °C storage, though this should have affected both subpopulations comparably. Sixth, assessment was restricted to a single center in one country, so regional confounders cannot be excluded. Seventh, machine-learning approaches carry risks of bias from black-box methodology and overfitting. Eight, lack of multiple testing correction (e.g., Bonferroni approach [60]), consistent with the exploratory, hypothesis-generating nature of the assessment, bears the risk of overinterpretation.

## 5. Conclusions

Despite the limitations outlined above, this study identified associations between *Shigella*/EIEC and EAEC with HIV-related immunosuppression, whereas the opposite pattern was observed for EPEC. Regarding clinically apparent diarrhea, the detected association patterns largely mirrored those described previously in Ghanaian and Tanzanian children, except for *Salmonella* spp., which showed a specific link with diarrhea in the present study population. The basis of the negative association between enteric EPEC abundance and HIV infection remains unclear. Future investigations are warranted to further assess interactions between immune status, pathogen abundance, and clinical manifestations of gastrointestinal infections.

## Figures and Tables

**Table 1:** Demographics, socioeconomic parameters, medical parameters, and immunological parameters in the study population according to HIV status.

	Variable	HIV-Positive Individuals, n = 651	HIV-Negative Individuals, n = 84
Demographics	Age in years, median (IQR)	40 (33/47)	29 (24/37)
	Female, n (%)	469 (73.63)	53 (64.63)
Socioeconomic parameters	Access to tap water, n (%)	337 (52.9)	53 (63.86)
	Electricity in household, n (%)	595 (93.41)	80 (96.39)
	Refrigerator in household, n (%)	463 (72.68)	68 (81.93)
	SES index high, n (%)	459 (72.06)	68 (81.93)
Medical parameters	Cotrimoxazole prophylaxis, n (%)	206 (33.01)	NA
	Intake of cART, n (%)	263 (41.29)	NA
	Body mass index, median (IQR)	22 (20/26)	24 (21/27)
Virological andimmunological parameters	Viral load, log10 copies/mL, median (IQR)	4.2 (1.6/5.4)	NA
	CD4+ T cell count/µL, median (IQR)	347 (145/571)	949 (767/1168)
	CD8+ T cell count/µL, median (IQR)	970 (648/1379)	470 (354/710)
	CD4+/CD8+ T cell ratio, median (IQR)	0.4 (0.2/0.7)	2.0 (1.6/2.5)

cART—antiretroviral combination therapy; IQR—Interquartile range; NA—not applicable; SES index—socioeconomical index.

**Table 2:** Correlation of cycle threshold (Ct) values of the real-time PCR assays targeting the assessed enteropathogens with CD4+ T-lymphocyte count.

Ct Values of the Real-Time PCR Assays		
	Spearman’s ρ	*p*-Value
*Arcobacter butzleri*	0.03	0.858
*Campylobacter jejuni*	−0.11	0.692
EAEC	0.10	0.018
EPEC (assay 1 targeting the *eae* gene)	0.05	0.249
EPEC (assay 2 targeting an EAF plasmid sequence)	−0.18	0.058
ETEC	−0.10	0.102
*Salmonella* spp.	0.19	0.078
*Shigella* spp.	0.18	0.025

**Table 3:** Comparison of diarrhea-associations of detected enteric microorganisms from the present study as well as from historic studies on children from Ghana and Tanzania [66,67].

Microorganism	Present Study on a Ghanaian Population with HIV	Ghanaian Children [66]	Tanzanian Children [67]
*Salmonella* spp.	Associated with diarrhea	No association with diarrhea detected	No association with diarrhea detected
*Shigella* spp./enteroinvasive *Escherichia coli*	Associated with diarrhea	Associated with diarrhea	No association with diarrhea detected *
*Campylobacter jejuni*	No association with diarrhea detected	No association with diarrhea detected	No association with diarrhea detected
enteropathogenic *Escherichia coli*	No association with diarrhea detected	Not assessed	No association with diarrhea detected
enterotoxigenic *Escherichia coli*	No association with diarrhea detected	Not assessed	No association with diarrhea detected
enteroaggregative *Escherchia coli*	No association with diarrhea detected	Not assessed	No association with diarrhea detected
*Arcobacter butzleri*	No association with diarrhea detected	Not assessed	Not assessed

* association with high bacterial load.

## Data Availability

The original contributions presented in this study are included in the article. Further inquiries can be directed to the corresponding author.

## Abbreviations

The following abbreviations are used in this manuscript:

95% CI	95% confidence interval
AIDS	acquired immunodeficiency syndrome
cART	combination antiretroviral therapy
Ct	cycle threshold
DNA	desoxyribonucleic acid
EAEC	enteroaggregative *Escherichia coli*
EIEC	enteroinvasive *Escherichia coli*
EPEC	enteropathogenic *Escherichia coli*
ETEC	enterotoxigenic *Escherichia coli*
HIV	human immunodeficiency virus
IQR	inter-quartile range
min.	minute
µL	microliter
N	number
NA	not applicable
PCA	principal component analysis
PCR	polymerase chain reaction
PLWH	people-living-with-HIV
SD	standard deviation
sec.	second
SES	socio-economic status
spp.	species (plural)

## Appendix A

**Table A1:** Target genes, calculated detection limits and oligonucleotides used for the applied real-time PCR screening assays for the assessed entero-invasive and non-entero-invasive diarrheagenic bacteria. Hyphens in the oligonucleotide sequences have been inserted to increase the readability, not to delineate codon triplets.

PCR target	*Salmonella* spp.
Target gene	*ttrC*
Detection limit	3.6 × 10^2^ copies/µL
Forward primer	5′-ATT-GTT-GAT-TCA-GGT-ACA-AAC-3′
Reverse primer	5′-AAT-TAG-CCA-TGT-TGT-AAT-CTC-3′
Probe and modifications	5′-JOE-CAA-GTT-CAA-CGC-GCA-ATT-TA-BHQ1-3′
Positive control plasmid insert	5′-CTG-GAC-ATT-GTT-GAT-TCA-GGT-ACA-AAC-CGT-CCC-CAA-GTT-CAA-CGC-GCA-ATT-TAA-CCC-TTA-CTC-GTT-ACC-AGG-CGG-AAC-GGA-TGG-CTG-GCT-GGC-TAT-TCT-CGG-CAC-CTT-CGG-CCT-GTG-GAT-AGC-GCT-ACT-GAT-TAT-TAT-TCG-TGA-AAC-GCT-GAA-CGG-ACT-CAC-CAG-GAG-ATT-ACA-ACA-TGG-CTA-ATT-TAA-CCC-3′
GenBank accession number used for the insert	CP007365.2
Reference	[55]
PCR target	*Shigella* spp./entero-invasive *Escherichia coli* (EIEC)
Target gene	*ipaH*
Detection limit	3.6 × 10^2^ copies/µL
Forward primer	5′-CAG-AAG-AGC-AGA-AGT-ATG-AG-3′
Reverse primer	5′-CAG-TAC-CTC-GTC-AGT-CAG-3′
Probe and modifications	5′-ROX-ACA-GGT-GAT-GCG-TGA-GAC-TG-BHQ2-3′
Positive control plasmid insert	5′-CGC-AGG-CAG-AAG-AGC-AGA-AGT-ATG-AGA-TGC-TGG-AGA-ATG-AGT-ACT-CTC-AGA-GGG-TGG-CTG-ACC-GGC-TGA-AAG-CAT-CAG-GTC-TGA-GCG-GTG-ATG-CGG-ATG-CGC-AGA-GGG-AAG-CCG-GTG-CAC-AGG-TGA-TGC-GTG-AGA-CTG-AAC-AGC-AGA-TTT-ACC-GTC-AGC-TGA-CTG-ACG-AGG-TAC-TGG-CCC-TG-3′
GenBank accession number used for the insert	M32063.1
Reference	[55]
PCR target	*Campylobacter jejuni*
Target gene	*gyrA*
Detection limit	3.6 × 10^2^ copies/µL
Forward primer	5′-CTA-TAA-CAA-CTG-CAC-CTA-CTA-AT-3′
Reverse primer	5′-ATG-AAA-TTT-TTG-CCA-GTG-GTG-3′
Probe and modifications	5′-FAM-CTT-AAT-AGC-CGT-CAC-CCC-AC-BHQ1-3′
Positive control plasmid insert	5′-*CAT-TTT-CTA-TAA-CAA-CTG-CAC-CTA-CTA-ATT-CGT-CAT-TTT-TCT-CTT-TAA-ACT-TAA-TAG-*CCG-TCA-CCC-CAC-GAC-TTA-CAC-GAC-CGA-TTT-CAC-GTA-CTT-TAG-CAA-GTG-GGA-ATT-TGA-TAC-ACA-TAC-CTT-TTT-TGG-TTA-CTG-CAA-AGA-GCA-TTT-TAC-CTT-GTG-TGC-TTA-CAC-TTT-CTT-CAT-TTT-CAA-GAT-TTT-CAT-CAT-CTA-AAT-TTT-CAA-TTT-CTT-GAT-TTT-CTA-AAT-TTT-CTT-CAC-CAC-CAC-TGG-CAA-AAA-TTT-CAT-CTT-CAT-3′
GenBank accession number used for the insert	CP012244.1
Reference	[55]
PCR target	Enteropathogenic *Escherichia coli* (EPEC) PCR 1
Target gene	*eae* gene
Detection limit	2.5 × 10^1^ copies/µL
Forward primer	5′-CAT-TGA-TCA-GGA-TTT-TTC-TGG-TGA-TA-3′
Reverse primer	5′-CTC-ATG-CGG-AAA-TAG-CCG-TTA-3′
Probe and modifications	5′-ROX-ATA-GTC-TCG-CCA-GTA-TTC-GCC-ACC-AAT-ACC-BHQ2-3′
Positive control plasmid insert	5′-CGT-CTT-CAT-TGA-TCA-GGA-TTT-TTC-TGG-TGA-TAA-TAC-CCG-TTT-AGG-TAT-TGG-TGG-CGA-ATA-CTG-GCG-AGA-CTA-TTT-CAA-AAG-TAG-TGT-TAA-CGG-CTA-TTT-CCG-CAT-GAG-CGG-CTG-3′
GenBank accession number used for the insert	Z11541.1
Reference	[54]
PCR target	Enteropathogenic *Escherichia coli* (EPEC) PCR 2
Target gene	EAF plasmid sequence
Detection limit	3.1 × 10^1^ copies/µL
Forward primer	5′-CAG-GGT-AAA-AGA-AAG-ATG-ATA-A-3′
Reverse primer	5′-GCA-TGG-AAC-ATC-GAT-CAG-TGA-3′
Probe and modifications	5′-6-FAM-TGG-AGT-GAT-CGA-ACG-GGA-TCC-A-BHQ1-3′
Positive control plasmid insert	5′-AAA-AAA-CAG-GGT-AAA-AGA-AAG-ATG-ATA-AGT-TAA-CGC-TTG-GAG-TGA-TCG-AAC-GGG-ATC-CAA-ATC-ACT-GAT-CGA-TGT-TCC-ATG-CGA-ATA-AGT-GAT-CGA-TCA-TGT-CGG-AAT-ATC-CAA-AAA-CCC-GAA-ATC-ACC-AGT-TGC-CAC-ATT-GAA-CGG-CGC-TGG-TGA-TTT-CGG-GTT-CGT-CAC-TTT-ATG-GAT-ACC-ATC-AAC-CCA-TTC-CCC-GGA-GAA-AGT-ATG-GGC-TGG-CTA-AAG-TGT-AGC-GNC-ATT-AAG-AGC-AGT-TAT-TTA-GTA-TTT-TAA-TGA-GTA-TCG-AAT-CTT-TAT-ATT-TGC-ATC-ATT-CCG-TTG-TTG-GTC-CGC-CTT-CTG-ACA-AGC-TGT-GTT-GGC-AGA-AGA-AAC-GTC-GTT-AGC-GGT-TCC-TAT-TTT-GTT-ACT-ACC-TAG-ATA-TAT-ATC-AGG-TTT-TTG-ATA-ATA-CAT-GGT-CCC-CAT-ATT-CAT-A-3′
GenBank accession number used for the insert	X76137.1
Reference	[54]
PCR target	Enterotoxigenic *Escherichia coli* (ETEC)
Target gene	*eltB* gene (component 1) and *estB* gene (component 2)
Detection limit	2.7 × 10^1^ copies/µL
Component	1
Forward primer	5′-GCG-TTA-CTA-TCC-TCT-CTA-TG-3′
Reverse primer	5′-TGA-TAT-TCC-GAA-CAT-AGT-TCT-GTA-3′
Probe and modifications	5′-JOE-TAG-ACT-GGG-GAG-CTC-CGT-GTG-C-BHQ1-3′
Positive control plasmid insert	5′-TAT-TTA-CGG-CGT-TAC-TAT-CCT-CTC-TAT-GTG-CAC-ACG-GAG-CTC-CCC-AGT-CTA-TTA-CAG-AAC-TAT-GTT-CGG-AAT-ATC-ACA-ACA-CAC-AAA-TAT-ATA-CGA-TAA-ATG-ACA-AGA-TAC-TAT-CAT-ATA-CGG-AAT-CGA-TGG-CAG-GCA-AAA-GAG-AAA-TGG-TTA-TCA-TTA-CAT-TTA-AGA-GCG-GCG-CAA-CAT-TTC-AGG-TCG-AAG-TCC-CGG-GCA-GTC-AAC-ATA-TAG-ACT-CCC-AAA-AAA-AAG-CCA-TTG-AAA-GGA-TGA-AGG-ACA-CAT-TAA-GAA-TCA-CAT-ATC-TGA-CCG-AGA-CCA-AAA-TTG-ATA-AAT-TAT-GTG-TAT-GGA-ATA-ATA-AAA-CCC-CCA-ATT-CAA-TT-3′
GenBank accession number used for the insert	KJ716874.1
Component	2
Forward primer	5′-TCC-CTC-AGG-ATG-CTA-AAC-3′
Reverse primer	5′-CAA-CAA-AGC-AAC-AGG-TAC-ATA-CGT-3′
Probe and modifications	5′-JOE-ATA-GCA-CCC-GGT-ACA-AGC-AGG-BHQ1-3′
Positive control plasmid insert	5′-CAC-CTT-TCC-CTC-AGG-ATG-CTA-AAC-CAG-TAG-AGT-CTT-CAA-AAG-AAA-AAA-TCA-CAC-TAG-AAT-CAA-AAA-AAT-GTA-ACA-TTG-CAA-AAA-AAA-GTA-ATA-AAA-GTG-GTC-CTG-AAA-GCA-TGA-ATA-GTA-GCA-ATT-ACT-GCT-GTG-AAT-TGT-GTT-GTA-ATC-CTG-CTT-GTA-CCG-GGT-GCT-ATT-AAT-AAT-ATA-AAG-GGA-ACT-AAA-CAG-TTC-CCT-TTA-TAT-TTG-TTC-TGA-TTC-TGA-TGA-TGT-CTG-TAA-CGT-ATG-TAC-CTG-TTG-CTT-TGT-TGA-ATA-AA-3′
GenBank accession number used for the insert	M34916.1
Reference	[54]
PCR target	Enteroaggregative *Escherichia coli* (EAEC)
Target gene	*aatA* gene
Detection limit	1.2 × 10^1^ copies/µL
Forward primer	5′-CAA-TGT-ATA-GAA-ATC-CGC-TGT-T-3′
Reverse primer	5′-CTG-TCA-GAT-AAA-ATC-TCG-AGA-GAA-3′
Probe and modifications	5′-Cy5-CATGTTCCTGAGAGTGCAATCCCAG-BHQ2-3′
Positive control plasmid insert	5′-AGC-TAA-TAA-TGT-ATA-GAA-ATC-CGC-TGT-TTT-ACA-CTC-TTT-TAA-CTT-ATG-ATA-TGT-AAT-GTC-TGG-GAT-TGC-ACT-CTC-AGG-AAC-ATG-ATA-TTC-TCT-CGA-GAT-TTT-ATC-TGA-CAG-TAA-ACT-TTC-CTC-CTC-CTC-AAG-GAC-A-3′
GenBank accession number used for the insert	X81423.1
Reference	[54]
PCR target	*Arcobacter butzleri*
Target gene	*rpoB/C*
Detection limit	3.7 × 10^2^ copies/µL
Forward primer	5′-GCC-ACA-CCA-GTG-ACA-ATA-TC-3′ & 5′-AAA-AAA-TAC-TTT-CTT-GGT-CTT-GTG-GTG-TA-3′
Reverse primer	5′-AAC-AAC-ACC-TTT-GTA-TCT-CAT-TTT-TTT-G-3′
Probe and modifications	5′-HEX-TTG-GAC-CAG-TAA-AAG-ATT-ATG-AGT-GTC-TTT-GTG-GTA-AA-BHQ1-3′
Positive control plasmid insert	5′-GCA-AGT-CCA-GAA-AAA-ATA-CTT-TCT-TGG-TCT-TGT-GGT-GAA-GTT-AAA-AAA-CCT-GAA-ACA-ATT-AAT-TAT-AGA-ACA-TTA-AAA-CCA-GAA-AGA-GAT-GGA-TTA-TTT-TGT-GCT-AAA-ATT-TTT-GGA-CCA-GTA-AAA-GAT-TAT-GAG-TGT-CTT-TGT-GGT-AAA-TAC-AAA-AAA-ATG-AGA-TAC-AAA-GGT-GTT-GTT-TGC-GAA-A-3′
GenBank accession number used for the insert	AB104468.1
Reference	[56,57]

spp. = species. Underlined bases = intended A-G-mismatches and C-T-mismatches to affect binding affinity and annealing temperature. EIEC = entero-invasive *Escherichia coli*, EAEC = enteroaggregative *E. coli*, ETEC = enterotoxigenic *E. coli*, EPEC = enteropathogenic *E. coli.*

**Table A2:** Reaction mixes and run conditions for the assessed entero-invasive and non-entero-invasive diarrheagenic bacteria.

	*Salmonella* spp., *Shigella* spp./EIEC, *Campylobacter jejuni* PCR	EPEC, ETEC, EAEC PCR	*Arcobacter butzleri* PCR
Reaction chemistry			
Master Mix	HotStar master mix (Qiagen, Hilden, Germany)	HotStar master mix (Qiagen, Hilden, Germany)	HotStar master mix (Qiagen, Hilden, Germany)
Reaction volume (µL)	20.0	20.0	20.0
Forward primer concentration (nM)	750.0 (*Salmonella* spp.), 375.0 (*Shigella* spp./EIEC), 125.0 (*C. jejuni*)	125.0	300.0
Reverse primer concentration (nM)	750.0 (*Salmonella* spp.), 375.0 (*Shigella* spp./EIEC), 125.0 (*C. jejuni*)	125.0	300.0
Probe concentration (nM)	42.0 (*Salmonella* spp.), 21.0 (*Shigella* spp./EIEC), 35.0 (*C. jejuni*)	18.0 (*eae* gene of EPEC), 35.0 (EAF plasmid of EPEC and aatA gene of EAEC), 70.0 (*estB* and *eltA* genes of ETEC)	100.0
Final Mg^2+^ concentration (mM)	3.0	6.0	4.5
Bovine serum albumin (ng/µL)	-	2.0	5.0
Eluate volume (µL)	2.0	2.0	2.0
Run conditions			
Initial denaturation	15 min. at 95 °C	15 min. at 95 °C	15 min. at 95 °C
Cycle numbers	45	45	40
Denaturation	15 sec. at 95 °C	15 sec. at 95 °C	15 sec. at 95 °C
Annealing	20 sec. at 56 °C with a touchdown 13 × 0.5/cycle	30 sec. at 60 °C	60 sec. at 60 °C
Amplification	30 sec. at 72 °C	30 sec. at 72 °C	together with annealing
Hold	30 sec. at 40 °C	20 sec. at 40 °C	20 sec. at 40 °C

min. = minute, sec. = second, spp. = species, EIEC = entero-invasive *Escherichia coli*, EAEC = enteroaggregative *E. coli*, ETEC = enterotoxigenic *E. coli*, EPEC = enteropathogenic *E. coli.*
